# Di-*n*-propyl 4,4′-dihy­droxy-3,3′-{[(3a*RS*,7a*RS*)-2,3,3a,4,5,6,7,7a-octa­hydro-1*H*-benzimidazole-1,3-di­yl]bis­(methyl­ene)}dibenzoate

**DOI:** 10.1107/S1600536811036385

**Published:** 2011-09-14

**Authors:** Augusto Rivera, Diego Quiroga, Jaime Ríos-Motta, Karla Fejfarová, Michal Dušek

**Affiliations:** a Departamento de Química, Universidad Nacional de Colombia, Ciudad Universitaria, Bogotá, Colombia; b Institute of Physics ASCR, v.v.i., Na Slovance 2, 182 21 Praha 8, Czech Republic

## Abstract

The title compound, C_29_H_38_N_2_O_6_, was prepared as model for studying intra­molecular hydrogen-bonding inter­actions. Mol­ecules of the title compound are located on a crystallographic twofold rotation axis, which passes through the C atom linked to the two N atoms on the imidazolidine ring. The mol­ecular structure shows the existence of two intra­molecular O—H⋯N hydrogen-bonding inter­actions between the two N atoms of the imidazolidine moiety and the hy­droxy groups in the aromatic rings. The crystal structure shows the strain of ring fusion in the perhydro­benzimidazole moiety according to the endocyclic bond angles and the torsion angles, which evidence a puckering of the cyclo­hexane ring with respect to normal tetra­hedral bond angles in an ideal chair conformation.

## Related literature

For a related structure, see: Rivera *et al.* (2010 ▶). For crystallographic data of *n*-propyl 4-hy­droxy­benzoate, see: Zhou *et al.* (2010 ▶); Feng & Grant (2006 ▶). For background chemistry to this work, see: Lu *et al.* (2006 ▶); Geise *et al.* (1971 ▶). For the synthesis of the precursor, see: Murray-Rust & Riddell (1975 ▶).
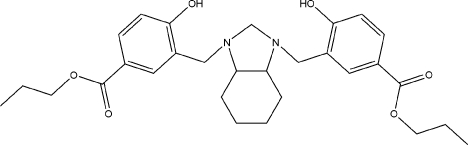


## Experimental

### 

#### Crystal data

C_29_H_38_N_2_O_6_
*M* *_r_* = 510.6
Monoclinic, 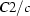

*a* = 15.8047 (4) Å
*b* = 8.7762 (3) Å
*c* = 19.0108 (6) Å
β = 96.353 (2)°
*V* = 2620.70 (14) Å^3^
*Z* = 4
Cu *K*α radiation
μ = 0.73 mm^−1^
*T* = 120 K
0.43 × 0.18 × 0.10 mm

#### Data collection

Agilent Gemini A Ultra diffractometer
Absorption correction: multi-scan (*CrysAlis PRO*; Agilent, 2010 ▶) *T*_min_ = 0.638, *T*_max_ = 1
18471 measured reflections
2339 independent reflections
1855 reflections with *I* > 3σ(*I*)
*R*_int_ = 0.044

#### Refinement

*R*[*F* ^2^ > 2σ(*F* ^2^)] = 0.039
*wR*(*F* ^2^) = 0.105
*S* = 1.57
2339 reflections
172 parameters
H atoms treated by a mixture of independent and constrained refinement
Δρ_max_ = 0.21 e Å^−3^
Δρ_min_ = −0.17 e Å^−3^

### 

Data collection: *CrysAlis PRO* (Agilent, 2010 ▶); cell refinement: *CrysAlis PRO*; data reduction: *CrysAlis PRO*; program(s) used to solve structure: *SIR2002* (Burla *et al.*, 2003 ▶); program(s) used to refine structure: *JANA2006* (Petříček *et al.*, 2006 ▶); molecular graphics: *DIAMOND* (Brandenburg & Putz, 2005 ▶); software used to prepare material for publication: JANA2006.

## Supplementary Material

Crystal structure: contains datablock(s) global, I. DOI: 10.1107/S1600536811036385/nk2109sup1.cif

Structure factors: contains datablock(s) I. DOI: 10.1107/S1600536811036385/nk2109Isup2.hkl

Supplementary material file. DOI: 10.1107/S1600536811036385/nk2109Isup3.cml

Additional supplementary materials:  crystallographic information; 3D view; checkCIF report

## Figures and Tables

**Table 1:** Hydrogen-bond geometry (Å, °)

*D*—H⋯*A*	*D*—H	H⋯*A*	*D*⋯*A*	*D*—H⋯*A*
O3—H3⋯N1	0.93 (2)	1.82 (2)	2.6810 (14)	153 (2)

**Fig. 1.**
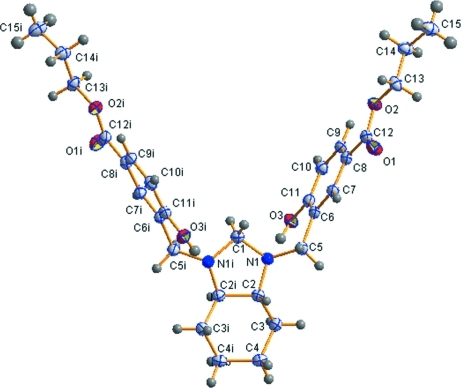
A view of (I). Displacement ellipsoids are drawn at the 50% probability level.

## supplementary crystallographic information

### Comment

Hydrogen bonding involving phenols has been the subject of extensive experimental and theoretical studies because hydrogen-bonding interactions of phenol itself can be regarded as a prototype to understand the attraction between the lone pair of the amine nitrogen atom and the phenolic hydroxyl proton. (Lu *et al.* 2006). Continuing our studies on the synthesis and structural analysis of Mannich bases derived from phenols, the title compound, (I), was obtained from *n*-propyl-4-hydroxybenzoate and (2*R*,7*R*,11*S*,16*S*)-1,8,10,17- tetraazapentacyclo[8.8.1.1^8,17^0^2,7^.0^11,16^]icosane.

The molecular structure and atom-numbering scheme for (I) are shown in Fig. 1. The six-membered ring exists in a chair conformation with a C2—C3—C4 [107.6 (1)°] bond angle which is slightly distorted respect to the normal tetrahedral bond angles in a ideal chair conformation [111.1°] (Geise, *et al.* 1971). These values suggest a constraint of the cyclohexane ring, which is minimized by an increasing of the C3—C4—C4^i^ bond angle [113.4 (1)°]. The imidazolidine moiety has a half-chair conformation (*C2*) with intraanular bond angles ranging from 105.1 (1)° to 106.6 (1)° which are shorter respect the tetrahedrical normal bond angles, indicating that the heterocyclic ring is also strained. This conformation is adopted because the nitrogen lone pairs are oriented *anti-axial* to avoid electronic repulsions. The bond length and bond angle values in the propoxycarbonyl group are in a good agreement with the values observed in the crystal structure of *n*-propyl 4-hydroxybenzoate (Feng & Grant, 2006; Zhou, *et al.* 2010).

Intramolecular hydrogen bonds are present between the phenolic hydroxyl groups and nitrogen atoms, the N···O distance [2.6810 (14) Å] is in a good agreement with the corresponding N···O distance in the phenol derivative [2.7096 (14) Å] (Rivera, *et al.* 2010).

### Experimental

The aminal (2*R*,7*R*,11*S*,16*S*)-1,8,10,17- tetraazapentacyclo[8.8.1.1^8,17^.0^2,7^.0^11,16^]icosane (276 mg, 1.00 mmol) prepared previously following described procedures (Murray-Rust & Riddell, 1975), was dissolved in dioxane (3 ml) at 70 °C with vigorous stirring. A solution of *n*-propyl 4-hydroxybenzoate (360 mg, 2.00 mmol) in dioxane (3 ml) was added dropwise for about 30 min, and then water (4 ml) was added. After the addition, the reaction mixture was refluxed for about 12 h. The reaction mixture was treated with chloroform by discontinuous liquid-liquid extraction (5 × 20 ml). The combined extracts were concentrated under reduced pressure until a residue appeared. The product was purified by chromatography on a silica column, and subjected to gradient elution with benzene:ethyl acetate (yield 19%, m.p. = 449–450 K).Single crystals of racemic (I) were grown from a CHCl_3_:MeOH solution by slow evaporation of the solvent at room temperature over a period of about two weeks.

### Refinement

All hydrogen atoms were discernible in difference Fourier maps and could be refined to reasonable geometry. According to common practice H atoms bonded to C atoms were kept in ideal positions with C–H distance 0.96 Å during the refinement. The methyl H atoms were allowed to rotate freely about the adjacent C—C bonds. The hydroxyl H atoms were found in difference Fourier maps and their coordinates were refined freely. All H atoms were refined with thermal displacement coefficients *U*_iso_(H) set to 1.5Ueq(C, O) for methyl and hydroxyl groups and to to 1.2Ueq(C) for the CH– and CH2- groups.

### Crystal data

**Table:**

C_29_H_38_N_2_O_6_	*F*(000) = 1096
*M**_r_* = 510.6	*D*_x_ = 1.294 Mg m^−^^3^
Monoclinic, *C*2/*c*	Cu *K*α radiation, λ = 1.5418 Å
Hall symbol: -C 2yc	Cell parameters from 6637 reflections
*a* = 15.8047 (4) Å	θ = 3.5–67.1°
*b* = 8.7762 (3) Å	µ = 0.73 mm^−^^1^
*c* = 19.0108 (6) Å	*T* = 120 K
β = 96.353 (2)°	Plate, colourless
*V* = 2620.70 (14) Å^3^	0.43 × 0.18 × 0.10 mm
*Z* = 4	

### Data collection

**Table:**

Agilent Gemini A Ultra diffractometer	2339 independent reflections
Radiation source: Enhance Ultra (Cu) X-ray Source	1855 reflections with *I* > 3σ(*I*)
mirror	*R*_int_ = 0.044
Detector resolution: 10.3784 pixels mm^-1^	θ_max_ = 67.2°, θ_min_ = 4.7°
Rotation method data acquisition using ω scans	*h* = −18→18
Absorption correction: multi-scan (*CrysAlis PRO*; Agilent, 2010)	*k* = −10→9
*T*_min_ = 0.638, *T*_max_ = 1	*l* = −22→22
18471 measured reflections	

### Refinement

**Table:**

Refinement on *F*^2^	H atoms treated by a mixture of independent and constrained refinement
*R*[*F*^2^ > 2σ(*F*^2^)] = 0.039	Weighting scheme based on measured s.u.'s *w* = 1/(σ^2^(*I*) + 0.0016*I*^2^)
*wR*(*F*^2^) = 0.105	(Δ/σ)_max_ = 0.010
*S* = 1.57	Δρ_max_ = 0.21 e Å^−^^3^
2339 reflections	Δρ_min_ = −0.17 e Å^−^^3^
172 parameters	Extinction correction: B-C type 1 Lorentzian isotropic (Becker & Coppens, 1974)
0 restraints	Extinction coefficient: 1100 (300)
73 constraints	

### Special details

**Table:**

Refinement. The refinement was carried out against all reflections. The conventional *R*-factor is always based on *F*. The goodness of fit as well as the weighted *R*-factor are based on *F* and *F*^2^ for refinement carried out on *F* and *F*^2^, respectively. The threshold expression is used only for calculating *R*-factors *etc*. and it is not relevant to the choice of reflections for refinement.The program used for refinement, Jana2006, uses the weighting scheme based on the experimental expectations, see _refine_ls_weighting_details, that does not force *S* to be one. Therefore the values of *S* are usually larger than the ones from the *SHELX* program.

### Fractional atomic coordinates and isotropic or equivalent isotropic displacement parameters (Å^2^)

**Table:**

	*x*	*y*	*z*	*U*_iso_*/*U*_eq_	
O1	0.44235 (7)	0.33457 (13)	0.45643 (6)	0.0351 (4)	
O2	0.34718 (6)	0.50352 (12)	0.48867 (5)	0.0273 (3)	
O3	0.29215 (7)	−0.02617 (13)	0.70432 (6)	0.0297 (4)	
N1	0.44367 (5)	−0.16285 (11)	0.70300 (4)	0.0242 (4)	
C1	0.5	−0.06183 (13)	0.75	0.0278 (7)	
C2	0.48251 (9)	−0.31564 (17)	0.71167 (8)	0.0242 (5)	
C3	0.42460 (9)	−0.45085 (18)	0.69460 (8)	0.0279 (5)	
C4	0.47714 (10)	−0.59581 (18)	0.71258 (8)	0.0293 (5)	
C5	0.43457 (10)	−0.11400 (18)	0.62864 (8)	0.0278 (5)	
C6	0.38601 (9)	0.03362 (18)	0.61649 (7)	0.0246 (4)	
C7	0.40693 (9)	0.13502 (18)	0.56533 (8)	0.0251 (5)	
C8	0.36156 (9)	0.26911 (18)	0.55025 (7)	0.0245 (5)	
C9	0.29302 (9)	0.30241 (17)	0.58796 (8)	0.0248 (5)	
C10	0.27134 (9)	0.20348 (18)	0.63976 (8)	0.0262 (5)	
C11	0.31679 (9)	0.06936 (18)	0.65395 (8)	0.0251 (4)	
C12	0.38833 (9)	0.36954 (18)	0.49413 (8)	0.0257 (5)	
C13	0.37042 (9)	0.59939 (18)	0.43146 (8)	0.0279 (5)	
C14	0.31759 (10)	0.74213 (19)	0.42731 (8)	0.0327 (5)	
C15	0.33797 (12)	0.8368 (2)	0.36391 (10)	0.0401 (6)	
H1a	0.533905	−0.000814	0.721944	0.0334*	
H2	0.52318	−0.329477	0.678263	0.029*	
H3a	0.40456	−0.450366	0.645051	0.0335*	
H3b	0.378108	−0.44685	0.723052	0.0335*	
H4a	0.440725	−0.683417	0.706234	0.0352*	
H4b	0.517975	−0.608248	0.679206	0.0352*	
H5a	0.406522	−0.192525	0.599622	0.0334*	
H5b	0.489912	−0.103289	0.612879	0.0334*	
H7	0.454193	0.112066	0.539465	0.0302*	
H9	0.260801	0.394109	0.577963	0.0297*	
H10	0.224629	0.227666	0.666045	0.0314*	
H13a	0.360587	0.544993	0.387508	0.0335*	
H13b	0.429613	0.625746	0.440121	0.0335*	
H14a	0.330535	0.800246	0.46993	0.0392*	
H14b	0.258305	0.715585	0.42174	0.0392*	
H15a	0.302902	0.926349	0.36018	0.0602*	
H15b	0.396838	0.866083	0.370217	0.0602*	
H15c	0.326969	0.77735	0.321488	0.0602*	
H3	0.3366 (14)	−0.095 (2)	0.7126 (10)	0.0446*	

### Atomic displacement parameters (Å^2^)

**Table:**

	*U*^11^	*U*^22^	*U*^33^	*U*^12^	*U*^13^	*U*^23^
O1	0.0361 (6)	0.0382 (7)	0.0322 (6)	0.0103 (5)	0.0094 (5)	0.0056 (5)
O2	0.0295 (5)	0.0255 (6)	0.0271 (6)	0.0032 (4)	0.0043 (4)	0.0048 (4)
O3	0.0301 (6)	0.0282 (7)	0.0311 (6)	0.0003 (5)	0.0038 (4)	0.0057 (5)
N1	0.0276 (6)	0.0206 (7)	0.0232 (7)	0.0008 (5)	−0.0025 (5)	−0.0003 (5)
C1	0.0318 (11)	0.0226 (12)	0.0277 (11)	0	−0.0024 (9)	0
C2	0.0267 (7)	0.0210 (8)	0.0245 (8)	0.0026 (6)	0.0013 (6)	0.0006 (6)
C3	0.0300 (8)	0.0246 (9)	0.0278 (8)	−0.0012 (6)	−0.0029 (6)	−0.0015 (6)
C4	0.0354 (8)	0.0203 (8)	0.0318 (9)	−0.0009 (6)	0.0009 (7)	−0.0023 (6)
C5	0.0340 (8)	0.0259 (9)	0.0226 (8)	0.0050 (7)	−0.0010 (6)	−0.0002 (6)
C6	0.0265 (7)	0.0237 (8)	0.0219 (7)	0.0026 (6)	−0.0047 (6)	−0.0034 (6)
C7	0.0261 (7)	0.0274 (9)	0.0213 (8)	0.0036 (6)	−0.0002 (6)	−0.0027 (6)
C8	0.0262 (7)	0.0247 (8)	0.0214 (7)	−0.0002 (6)	−0.0026 (6)	−0.0018 (6)
C9	0.0244 (7)	0.0219 (8)	0.0267 (8)	0.0025 (6)	−0.0029 (6)	−0.0033 (6)
C10	0.0228 (7)	0.0280 (9)	0.0276 (8)	0.0001 (6)	0.0018 (6)	−0.0021 (6)
C11	0.0265 (7)	0.0258 (9)	0.0219 (7)	−0.0032 (6)	−0.0021 (6)	−0.0010 (6)
C12	0.0267 (7)	0.0265 (9)	0.0226 (8)	0.0024 (6)	−0.0027 (6)	−0.0002 (6)
C13	0.0270 (7)	0.0306 (9)	0.0263 (8)	−0.0012 (6)	0.0036 (6)	0.0071 (7)
C14	0.0389 (9)	0.0289 (9)	0.0313 (9)	0.0029 (7)	0.0087 (7)	0.0033 (7)
C15	0.0486 (10)	0.0339 (10)	0.0400 (10)	0.0082 (8)	0.0147 (8)	0.0097 (8)

### Geometric parameters (Å, °)

**Table:**

O1—C12	1.2134 (19)	C5—H5b	0.96
O2—C12	1.3422 (19)	C6—C7	1.385 (2)
O2—C13	1.4539 (19)	C6—C11	1.405 (2)
O3—C11	1.3615 (19)	C7—C8	1.391 (2)
O3—H3	0.93 (2)	C7—H7	0.96
N1—C1	1.4835 (11)	C8—C9	1.395 (2)
N1—C2	1.4763 (17)	C8—C12	1.481 (2)
N1—C5	1.4689 (17)	C9—C10	1.384 (2)
C1—H1a	0.96	C9—H9	0.96
C1—H1a^i^	0.96	C10—C11	1.390 (2)
C2—C2^i^	1.500 (2)	C10—H10	0.96
C2—C3	1.512 (2)	C13—C14	1.503 (2)
C2—H2	0.96	C13—H13a	0.96
C3—C4	1.537 (2)	C13—H13b	0.96
C3—H3a	0.96	C14—C15	1.527 (3)
C3—H3b	0.96	C14—H14a	0.96
C4—C4^i^	1.523 (2)	C14—H14b	0.96
C4—H4a	0.96	C15—H15a	0.96
C4—H4b	0.96	C15—H15b	0.96
C5—C6	1.511 (2)	C15—H15c	0.96
C5—H5a	0.96		
			
C12—O2—C13	113.86 (12)	C7—C6—C11	118.18 (14)
C11—O3—H3	104.6 (13)	C6—C7—C8	122.04 (14)
C1—N1—C2	105.14 (8)	C6—C7—H7	118.9808
C1—N1—C5	113.18 (9)	C8—C7—H7	118.9799
C2—N1—C5	111.58 (10)	C7—C8—C9	118.90 (14)
N1—C1—N1^i^	106.60 (9)	C7—C8—C12	118.03 (13)
N1—C1—H1a	109.4712	C9—C8—C12	123.07 (14)
N1—C1—H1a^i^	109.4713	C8—C9—C10	120.12 (14)
N1^i^—C1—H1a	109.4713	C8—C9—H9	119.9408
N1^i^—C1—H1a^i^	109.4712	C10—C9—H9	119.9415
H1a—C1—H1a^i^	112.196	C9—C10—C11	120.42 (14)
N1—C2—C2^i^	102.26 (11)	C9—C10—H10	119.7917
N1—C2—C3	117.04 (11)	C11—C10—H10	119.7918
N1—C2—H2	109.9711	O3—C11—C6	121.21 (13)
C2^i^—C2—C3	110.96 (12)	O3—C11—C10	118.45 (13)
C2^i^—C2—H2	116.1607	C6—C11—C10	120.34 (14)
C3—C2—H2	101.0977	O1—C12—O2	122.85 (14)
C2—C3—C4	107.61 (12)	O1—C12—C8	123.42 (14)
C2—C3—H3a	109.4708	O2—C12—C8	113.73 (13)
C2—C3—H3b	109.4712	O2—C13—C14	109.68 (13)
C4—C3—H3a	109.4711	O2—C13—H13a	109.4707
C4—C3—H3b	109.4719	O2—C13—H13b	109.4714
H3a—C3—H3b	111.2673	C14—C13—H13a	109.4706
C3—C4—C4^i^	113.37 (13)	C14—C13—H13b	109.4711
C3—C4—H4a	109.4715	H13a—C13—H13b	109.2656
C3—C4—H4b	109.4718	C13—C14—C15	109.29 (14)
C4^i^—C4—H4a	109.4713	C13—C14—H14a	109.4708
C4^i^—C4—H4b	109.4702	C13—C14—H14b	109.4715
H4a—C4—H4b	105.271	C15—C14—H14a	109.4706
N1—C5—C6	113.04 (12)	C15—C14—H14b	109.4717
N1—C5—H5a	109.4711	H14a—C14—H14b	109.6526
N1—C5—H5b	109.4709	C14—C15—H15a	109.4714
C6—C5—H5a	109.4723	C14—C15—H15b	109.4714
C6—C5—H5b	109.471	C14—C15—H15c	109.4713
H5a—C5—H5b	105.6475	H15a—C15—H15b	109.4713
C5—C6—C7	120.11 (13)	H15a—C15—H15c	109.4713
C5—C6—C11	121.66 (13)	H15b—C15—H15c	109.4708
			
?—?—?—?	?		

Symmetry codes: (i) −*x*+1, *y*, −*z*+3/2.

### Hydrogen-bond geometry (Å, °)

**Table:**

*D*—H···*A*	*D*—H	H···*A*	*D*···*A*	*D*—H···*A*
O3—H3···N1	0.93 (2)	1.82 (2)	2.6810 (14)	153 (2)
